# Beta tricalcium phosphate, either alone or in combination with antimicrobial photodynamic therapy or doxycycline, prevents medication-related osteonecrosis of the jaw

**DOI:** 10.1038/s41598-022-20128-4

**Published:** 2022-10-03

**Authors:** Henrique Hadad, Laís Kawamata de Jesus, Ana Flávia Piquera Santos, Henrique Rinaldi Matheus, Letícia Gabriella de Souza Rodrigues, Pier Paolo Poli, Elcio Marcantonio Junior, Fernando Pozzi Semeghini Guastaldi, Carlo Maiorana, Juliano Milanezi de Almeida, Roberta Okamoto, Francisley Ávila Souza

**Affiliations:** 1 Department of Diagnosis and Surgery, School of Dentistry, São Paulo State University (UNESP), 1193, José Bonifácio St, Vila Mendonça, Araçatuba, São Paulo 16015-050 Brazil; 2 Department of Biomedical, Surgical and Dental Sciences, Implant Center for Edentulism and Jawbone Atrophies, Maxillofacial Surgery and Odontostomatology Unit, Fondazione IRCCS Cá Granda Ospedale Maggiore Policlinico, University of Milan, Milan, Italy; 3 Department of Diagnosis and Surgery, School of Dentistry, São Paulo State University (UNESP), Araraquara, Brazil; 4 Skeletal Biology Research Center, Department of Oral and Maxillofacial Surgery, Massachusetts General Hospital (MGH), Harvard School of Dental Medicine, Boston, MA USA; 5 Department of Basic Sciences, School of Dentistry, São Paulo State University (UNESP), Araçatuba, Brazil

**Keywords:** Pathogenesis, Risk factors, Medical research, Experimental models of disease

## Abstract

Surgical trauma in those under a prolonged use of bisphosphonates, can lead to mediation-related osteonecrosis of the jaw (MRONJ). This study aimed to evaluate the preventive therapies for MRONJ. Following four cycles of zoledronic acid administration, *Wistar* rats had their molar extracted, and were organized into nine treatment groups: negative control group (NCG), treated with saline solution and blood-clot in the alveolus; positive control group (PCG), with blood-clot in the alveolus; BG, β-tricalcium phosphate-based biomaterial; DG, 10% doxycycline gel; aG, antimicrobial photodynamic therapy; and DBG, aBG, aDG, and aDBG, using combination therapy. After 28 days, the lowest bone volume (BV/TV) was reported in PCG (42.17% ± 2.65), and the highest in aDBG (69.85% ± 6.25) (*p* < 0.05). The higher values of daily mineral apposition rate were recorded in aDBG (2.64 ± 0.48) and DBG (2.30 ± 0.37) (*p* < 0.001). Moreover, aDBG presented with the highest neoformed bone area (82.44% ± 2.69) (*p* < 0.05). Non-vital bone was reported only in the PCG (37.94 ± 18.70%). Owing to the key role of the biomaterial, the combination approach (aDBG) was the most effective in preventing MRONJ following tooth extraction.

## Introduction

Anti-resorptive drugs (ARs), including anti-RANKL antibodies inhibit osteoclastic activity, and are thus commonly used to prevent hypercalcemia or bone metastases in patients with cancer^1,2^, and fractures in osteopenic/osteoporotic patients^3^. However, the prolonged use of these medications is a risk factor for medication-related osteonecrosis of the jaw (MRONJ)^4–7^. The prevalence of MRONJ is higher with the use of high bisphosphonate (BP) doses, especially of nitrogen-containing bisphosphonates (N-BPs), as they are more resistant to enzymatic degradation^8,9^.

According to a recent by the American Association of Oral and Maxillofacial Surgeons (AAOMS) position paper^9^, MRONJ is defined as an area of exposure in the maxillofacial region, persistent for > 8 weeks, that can be diagnosed in patients undergoing to antiresorptive therapy alone or in combination with immune modulators or antiangiogenic medications, without prior exposure to radiotherapy in the head and neck region^10–14^. Despite its advantages and benefits in the treatment of skeletal disorders, prolonged use of BPs can lead to the development of MRONJ^13,15,16^.

There is currently no universal protocol for the treatment of MRONJ. Treatment can range from conservative therapy to surgical intervention, depending on the disease stage. Several patients undergo conservative treatment, including oral health maintenance, infection removal, and antibiotic use^17^. Numerous published reports have described the success of antimicrobial photodynamic therapy (aPDT) in clinically treating this condition^18–22^.

Thus, studies exploring the prevention of MRONJ have emerged^23–25^. Recent in vitro studies have demonstrated the use of calcium phosphate ceramics in the reduction or elimination of zoledronic acid (ZOL) toxicity in fibroblasts^26^. In antimicrobial photodynamic therapy (aPDT), a low-power laser biostimulates the osteoblasts, even during ZOL treatment^27^, and reduces the number of bacterial colonies with a photosensitizing agent^24^. Moreover, satisfactory results were observed with doxycycline-loaded collagen sponge placed over the alveolar bone, in rats undergoing antiresorptive drug treatment^28^.

Thus, this study aimed to comparatively evaluate the preventive effects of locally applied β-tricalcium phosphate (BTCP) ceramics, aPDT, and doxycycline, alone or in combination, on the development of MRONJ, in the alveolar bone of rats undergoing ZOL therapy.

## Methods

This study was approved by the Ethics Committee on Animal Experimentation (CEUA) of São Paulo State University (UNESP), School of Dentistry, Araçatuba, Brazil (#0810-2018), following the Animal Research: Reporting of In Vivo Experiments (ARRIVE) guidelines, and in accordance with the Guide for the Care and Use of Laboratory Animals of the National Institutes of Health (Institute of Laboratory Animal Resources [U.S.])^29^.

### Animals

Seventy-two 3-month-old male Wistar rats (*Rattus norvegicus albinus*) (weight, 300–350 g), obtained from the UNESP facility were group-housed in cages (four animals per box), and acclimatized to a temperature of 24 ± 2 °C, with controlled light cycle (12/12 h), and free access to water and feed. To determine the sample size, a power test was performed at a significance level of 5% (with a standard deviation of 2%), α = 0.05, and 80% power. Thus, eight rats (n = 8) were required per group.

### ZOL application

All animals were treated with Zoledronate (ZOL) (Zometa®, Novartis Biosciences SA, São Paulo, Brazil), except for the animals in the negative control group (NCG), which received only 0.1 mL of saline solution (0.9% NaCl), using the same application protocol. Following an experimental model proposed by Curra et al.^30^ the animals received four intravenous (IV) applications of 0.035 mg/kg of ZOL, dissolved in 0.1 mL of vehicle (0.9% NaCl), through the tail vein, at 15-day intervals. One week after the fourth ZOL application, the mandibular right first molars of the animals were extracted, and locally treated. ZOL injections were continued until euthanasia.

### Tooth extraction and experimental groups

All animals had the mandibular right first molars extracted^23^ on the 52nd day of ZOL treatment initiation (corresponding to 7 days after the fourth ZOL application). The surgical procedure was performed under anesthesia via an intraperitoneal injection (IP) of 90 mg/kg of ketamine hydrochloride (Vetaset, Fort Dodge Animal Health Ltd., São Paulo, Brazil) and 10 mg/kg of xylazine hydrochloride (Dopaser, Laboratórios Calier do Brasil Ltda, São Paulo, Brazil). After sedation, antisepsis was performed with degerming and topical povidone-iodine (10% PVP-I; Riodeine, Rioquímica, São José do Rio Preto, Brazil) application, followed by sterile draping. The animals were placed in the dorsal decubitus position on a customized operating table, and syndesmotomy was carefully performed using a delicate detacher (Molt Descolador no. 9, Quinelato, São Paulo, Brazil), followed by luxation and extraction of the tooth. The animals were randomly divided into nine treatment groups (www.randomization.com) by a blinded assessor, unrelated to the study.

- **Negative Control group (NCG):** Treated systemically with NaCl and locally left empty, except for the blood clots in the alveolus.
- **Positive control group (PCG):** Treated systemically with ZOL and locally left empty, except for the blood clots in the alveolus.
- **Biomaterial group (BG):** The alveolus in the mandibular right first molar was filled with approximately 0.03 cc of BTCP paste (Graftys HBS, Latin American Solutions [LAS], Brazil).
- **Doxycycline group (DG):** The alveolus in the mandibular right first molar was filled with approximately 0.03 cc of 10% doxycycline gel.
- **aPDT group (aG):** The alveolus in the mandibular right first molar was irrigated for 1 min with 1 mL of neutral methylene blue photosensitizer, followed by a diode laser of indium gallium aluminum phosphide at the red wavelength (660 ± 10 nm), with a spot size of 0.0283 mm, 35 mW, in continuous mode, with a 2.1 J/point for 60 s, according to the model proposed by Ervolino et al.^24^.
- **Doxycycline + biomaterial group (DBG):** The alveolus in the mandibular right first molar was filled with approximately 0.03 cc of mixture in a proportion of 50% each (10% doxycycline gel and B-tricalcium phosphate paste, 1:1) (Graftys HBS, LAS, Brazil).
- **aPDT + biomaterial group (aBG):** The alveolus in the mandibular right first molar received aPDT (as described above) and was subsequently filled with approximately 0.03 cc of BTCP paste (Graftys HBS, LAS, Brazil).
- **aPDT + doxycycline group (aDG):** The alveolus in the mandibular right first molar received aPDT (as described above) and was subsequently filled with approximately 0.03 cc of 10% doxycycline gel.
- **aPDT + doxycycline + biomaterial group (aDBG):** The alveolus in the mandibular right first molar received aPDT (as described above) and was subsequently filled with approximately 0.03 cc of mixture in a proportion of 50% each (10% doxycycline gel and BTCP paste, 1:1) (Graftys HBS, LAS, Brazil).

Post-extraction, the soft tissue was detached from the extraction site, and sutured with 4–0 polyglactin 910 (Vicryl, Ethicon, Johnson Prod., São José dos Campos, Brazil), to assist in the clot maintenance, treatment as previously randomized, and primary closure of the alveolus.

### Fluorochrome application

Fifteen days after molar extraction, the fluorochrome calcein was administered intramuscularly (20 mg/kg) to each animal, and repeated after 10 days, using alizarin red (30 mg/kg)^31–33^.

### Euthanasia

**Figure 1:**
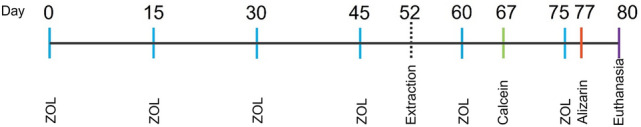
Flowchart of the work phases for mineralized tissue analysis, mentioned in days. (Blue lines, ZOL application; dotted line, extraction, and treatment of alveolus; green line, calcein application; red line, alizarin application; purple line, euthanasia, and collection of right hemimandible).

All animals were euthanized 80 days after ZOL application (28 days after molar extraction) with a lethal dose of sodium thiopental (150 mg/kg; Thiopentax, Cristália Ltda, Itapira, SP, Brazil) and IV administration of 2% injectable lidocaine hydrochloride (VETOne®; Pasadena, ID, USA). The right hemimandibles of all animals were collected and analyzed for the following; Fig. 1 shows the working scheme of the study.

### Microcomputed tomography analysis

The hemimandibles of the animals from the nine experimental groups were fixed in a 10% buffered formalin solution (Analytical Reagents, Dinâmica Odonto-Hospitalar Ltda, Catanduva, SP, Brazil) for 24 h, followed by washing under running water for 24 h. After fixation, the samples were stored in 70% alcohol for microcomputed tomography (micro-CT) analysis.

Micro-CT analysis was performed according to the methodology described by Bouxsein et al. (2010), using a SkyScan microtomograph (SkyScan 1272 Bruker MicroCT, Aatselaar, Belgium, 2003)^34^. The samples were scanned in 6-µm thick Sects. (90 kV and 111 μA), with a 0.5 mm Al + 0.038 mm Cu filter, and a 0.5-mm rotation step, at a pixel size of 2,016 × 1,344 µm, and acquisition time of 52 min. The distal root of the mandibular first molar was established as the region of interest (ROI), and a circular ROI size of 200 was used for all the specimens. The X-ray projection images of the samples were stored and reconstructed, and the area of interest was determined using the NRecon software (version 1.6.6.0; SkyScan, 2011), with the following modes: smoothing, 1; correction of artifact rings, 8; correction, 24% beam hardening, and image conversion, ranging from 0.0 to 0.14. The images were reconstructed in three planes (transverse, longitudinal, and sagittal), using the DataViewer software (version 1.4.4, 64-bit; SkyScan). The percentage of bone volume (BV/TV), trabecular bone thickness (TbTh), separation and number of trabeculae (TbSp and TbN), and total porosity (PoTot) were evaluated using the CT-Analyser software (version 1.12.4.0; 2003-11SkyScan, 2012 Bruker MicroCT), and subsequently reconstructed in three dimensions (3D), using the CTVox software (version 2.7; SkyScan).

### Laboratory processing

After micro-CT analysis, the hemimandibles of the nine experimental groups were dehydrated using increasing concentrations of alcohol (70%, 90%, and 100%), and subsequently immersed at different concentrations (70/30, 50/50, 30/70, 0/100, and 0/100) of Technovit® light-curing resin (Heraeus Kulzer GmbH, Wehrheim, Germany), in a mixture with 100% alcohol solution. The samples were stabilized on a glass plate with composite resin, such that the long axis of the distal alveolus of the mandibular first molar was parallel to the ground. Subsequently, the Technovit resin was incorporated, light-cured, Exakt microtome-cut (Apparatebau GmbH, Hamburg, Germany), calcified, and ground using an Exakt system sander, until the center of the mesial root of the mandibular right second molar was located (standardized to establish the center of the alveolus of the first molar). Longitudinal sections of approximately 50-μm thickness, were obtained in the alveolar region (in the mesiodistal plane).

### Laser confocal microscopy

These sections were captured using a Leica CTR 4000 CS SPE confocal laser microscope (Leica Microsystems, Heidelberg, Germany) with a 10 × at the Confocal Fluorescence Microscopy Laboratory of the São Paulo State University (UNESP), School of Dentistry, Araraquara, Brazil. Thus, images of the fluorochromes calcein and alizarin red (old/new bone) were obtained separately, and finally reconstructed with an overlap, to assess bone turnover through the mineral apposition rate (MAR). The area of fluorochrome precipitation (calcein/alizarin) was measured using ImageJ software (National Institutes of Health, Bethesda, Maryland, USA). MAR was determined using the value obtained on tracing five measurements extending from the outer margin of calcein towards the outer margin of alizarin, and dividing it by 10, which represents the time interval between the two injections^35^.

### Histomorphometric analysis

After confocal microscopy analysis, all the slides were stained with Stevenel’s blue and acid fuchsin. Images of the sections, obtained at 1.6 × and 40 × magnifications, were analyzed under an optical light microscope (Diastar, Leica Reichert and Jung Products, Germany), and captured using an attached digital camera (Leica Microsystems DFC-300-FX, Germany), with 1.3 MP resolution. Histomorphometric analyses were performed using the ImageLab 2000 analysis software (version 2.4, Image Laboratory Inc., Belmont, MA, USA). The measurements obtained in micrometers (µm), were presented after conversion as percentage of the neoformed bone area (%NBA), connective tissue, and necrotic bone area (non-vital bone).

#### Statistical analysis

Data were analyzed using SigmaPlot (version 12.0; Exakt Graph and Data Analysis, USA). After performing a normality test (Shapiro–Wilk test) to determine the data distribution, a one-way analysis of variance test (ANOVA) was performed to verify the difference between the means of the groups, followed by Tukey’s post hoc test for multiple comparisons.

## Results

### Micro-CT analysis

#### Bone volume percentage (BV/TV)

**Figure 2:**
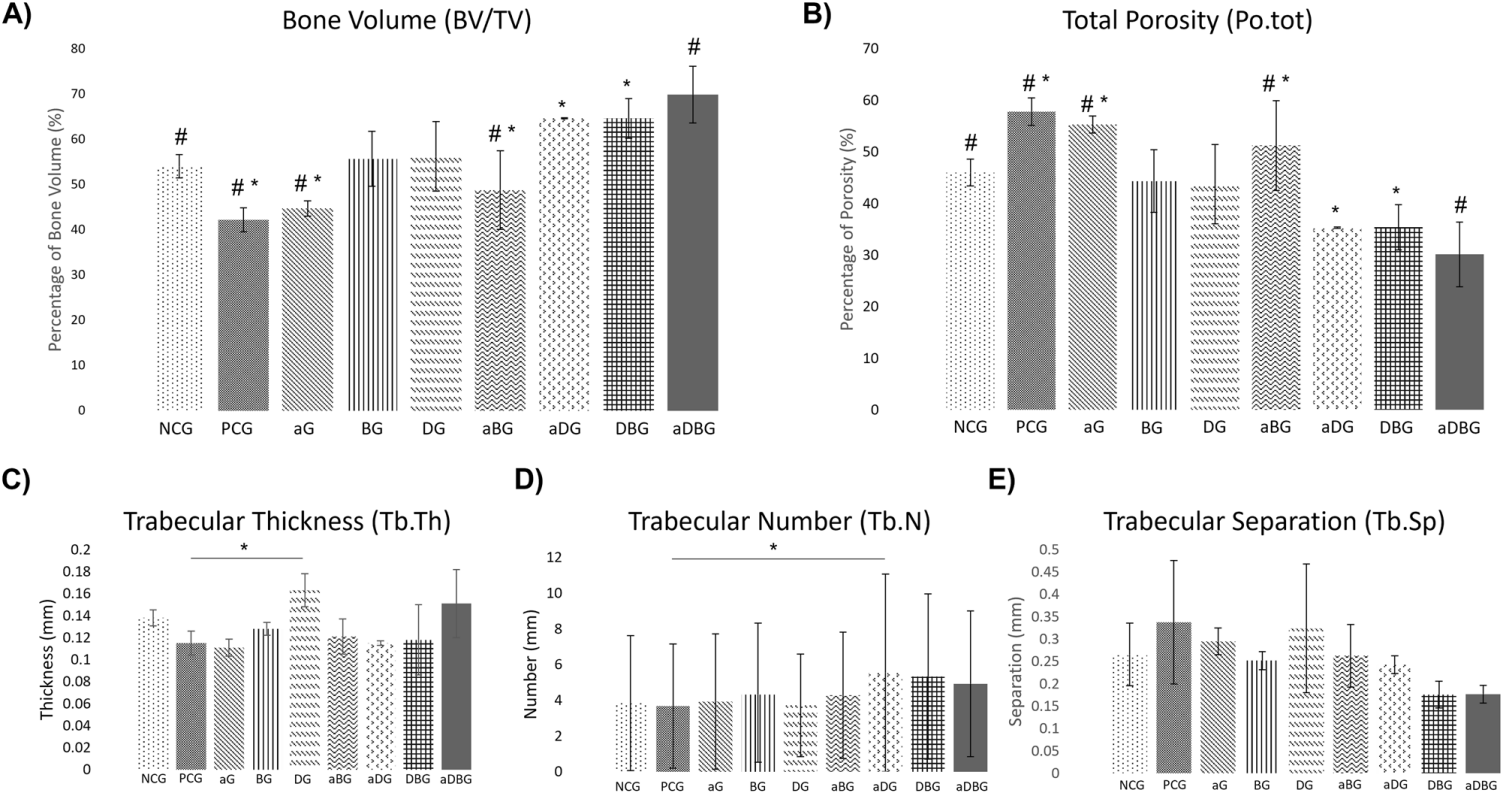
Representative images of the quantitative analysis of bone quality parameters obtained through micro-CT. (**A**) Representative image of the average percentage of bone volume in all groups (*p* < 0.05, represented by #, when compared to aDBG; *, when compared to aDG and DBG); (**B**) Image representing the average percentage of general porosity in all groups (*p* < 0.05, represented by #, when compared to aDBG; *, when compared to aDG and DBG); (**C**) Image representing the mean thickness of the bone trabeculae (in mm) in all groups (*, *p* < 0.05); (**D**) Image representing the mean number of trabeculae per mm in all groups (*p* > 0.05); (**E**) Image of the separation of trabeculae per mm in all groups (*p* > 0.05).

The NCG had an average BV/TV of 54.01% ± 2.60, whereas the PCG had an average BV/TV of 42.17% ± 2.65 (*p* = 0.193). Notably, all treatment groups exhibited a higher BV/TV than that of PCG (Fig. 2A). The aDBG had the highest BV/TV (69.85% ± 6.25), which was significantly different from that of NCG, PCG, aG, and aDG (*p* = 0.034, *p* < 0.001, *p* < 0.001 and, *p* = 0.003, respectively). However, no difference was observed when compared to BG, DG, aDG, and DBG (*p* = 0.071, *p* = 0.092, *p* = 0.941, and *p* = 0.939, respectively). The aDG and DBG presented with similar BV/TV values (64.62% ± 0.13 and 64.69% ± 4.40, respectively), significantly different from that of PCG, aG, and aBG (*p* = 0.002, 0.005, and 0.034, respectively).

#### Total porosity (PoTot)

The NCG had an average PoTot of 45.98% ± 2.60, whereas the PCG presented the highest value of 57.82% ± 2.65 (*p* = 0.193), which was significantly different from that of aDBG, aDG, and DBG (*p* < 0.001, *p* = 0.001, and *p* = 0.002, respectively), followed by aG with 55.31% ± 1.67, which was also significantly different when compared to aDBG, aDG, and DBG (*p* < 0.001, *p* = 0.005, and *p* = 0.005, respectively) (Fig. 2B).

#### Trabecular thickness (TbTh)

The NCG presented with a mean TbTh of 0.138 ± 0.0074 mm, whereas PCG had a mean TbTh of 0.115 mm ± 0.016 (*p* = 0.792). A significant difference (*p* = 0.043) was noted between DG (0.163 ± 0.015 mm) and aG (0.111 ± 0.0070 mm) (Fig. 2C).

#### Trabecular number (TbN)

A significant difference (*p* < 0.05) was observed between aDG (5.53 ± 5.3 mm) and PCG (3.68 ± 3.48 mm) (Fig. 2D).

#### Trabecular separation (TbSp)

In contrast to BV/TV, the aDBG, aDG, and DBG, which presented with the highest bone volume, in this analysis, demonstrated the lowest TbSp (0.177 ± 0.05 mm, 0.176 ± 0.03 mm, and 0.243 ± 0.02 mm, respectively); however, all groups remained within the general average (Fig. 2E).

#### Qualitative analysis of the alveolus

**Figure 3:**
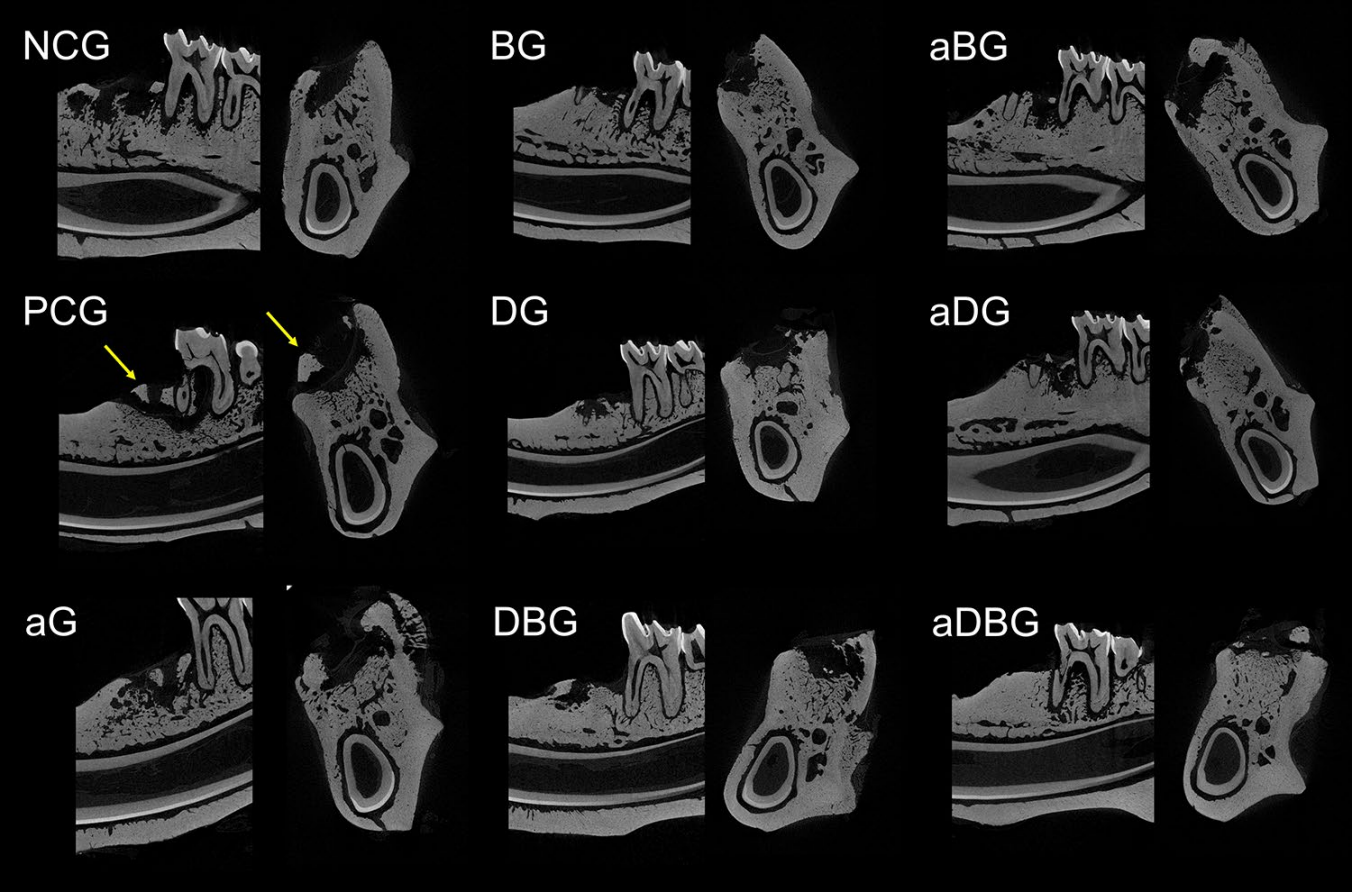
Representative images from the micro-CT. Each group is identified by its initials, followed by the sagittal and coronal section in the alveolar region of the distal root of the mandibular first molar. Yellow arrows demonstrate bone sequestrum.

Micro-CT images (Fig. 3) revealed a normal alveolar healing process, with mature, trabecular bone formation in the apical and middle thirds, thus maintaining the alveolar crest in the NCG. Conversely, in the PCG, bone sequestrum prevented the assessment of alveolar characteristics. Osteonecrosis was prevented in all the treatment groups, thus facilitating alveolar healing, and maintaining the alveolar anatomy. Furthermore, the alveolar volume was maintained in the BG, DG, and combination therapy groups (aDG, DBG, aBG, and aDBG), with aDBG exhibiting the most satisfactory results.

### Laser confocal microscopy

#### Mineral apposition rate

**Figure 4:**
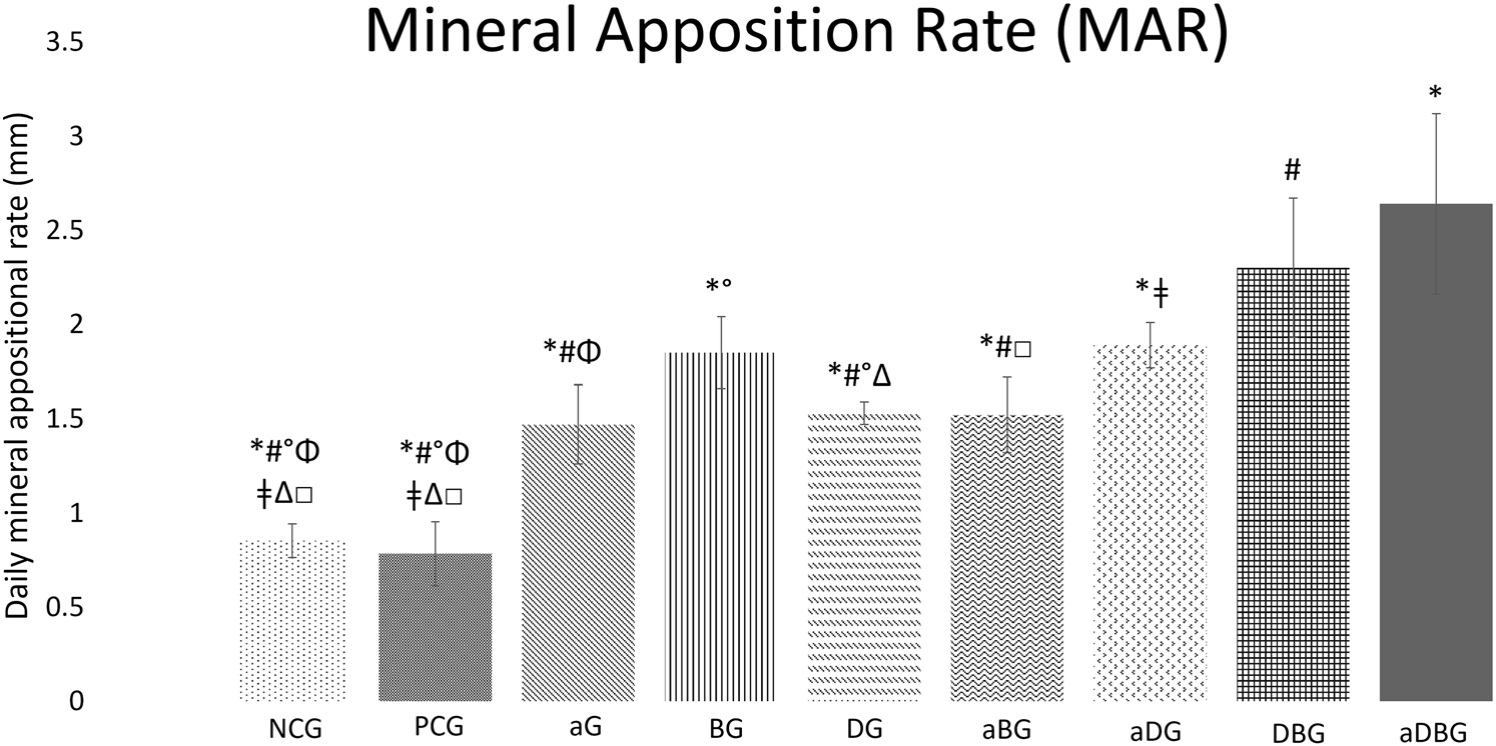
Graph representing the daily group averages of bone mineralization (*p* < 0.001, represented by *, when compared to aDBG; #, when compared to DBG; ǂ, when compared to aDG; °, when compared to BG; *p* < 0.05, represented by Δ, when compared to DG; □, when compared to aBG; Φ, when compared to aG).

The aDBG had the highest daily MAR (2.64 ± 0.48), when compared to the other groups (*p* < 0.001), except for DBG (2.30 ± 0.37, *p* = 0.359). All groups had higher MAR values, when compared to NCG and PCG (*p* < 0.05) (Fig. 4).

#### Qualitative analysis

**Figure 5:**
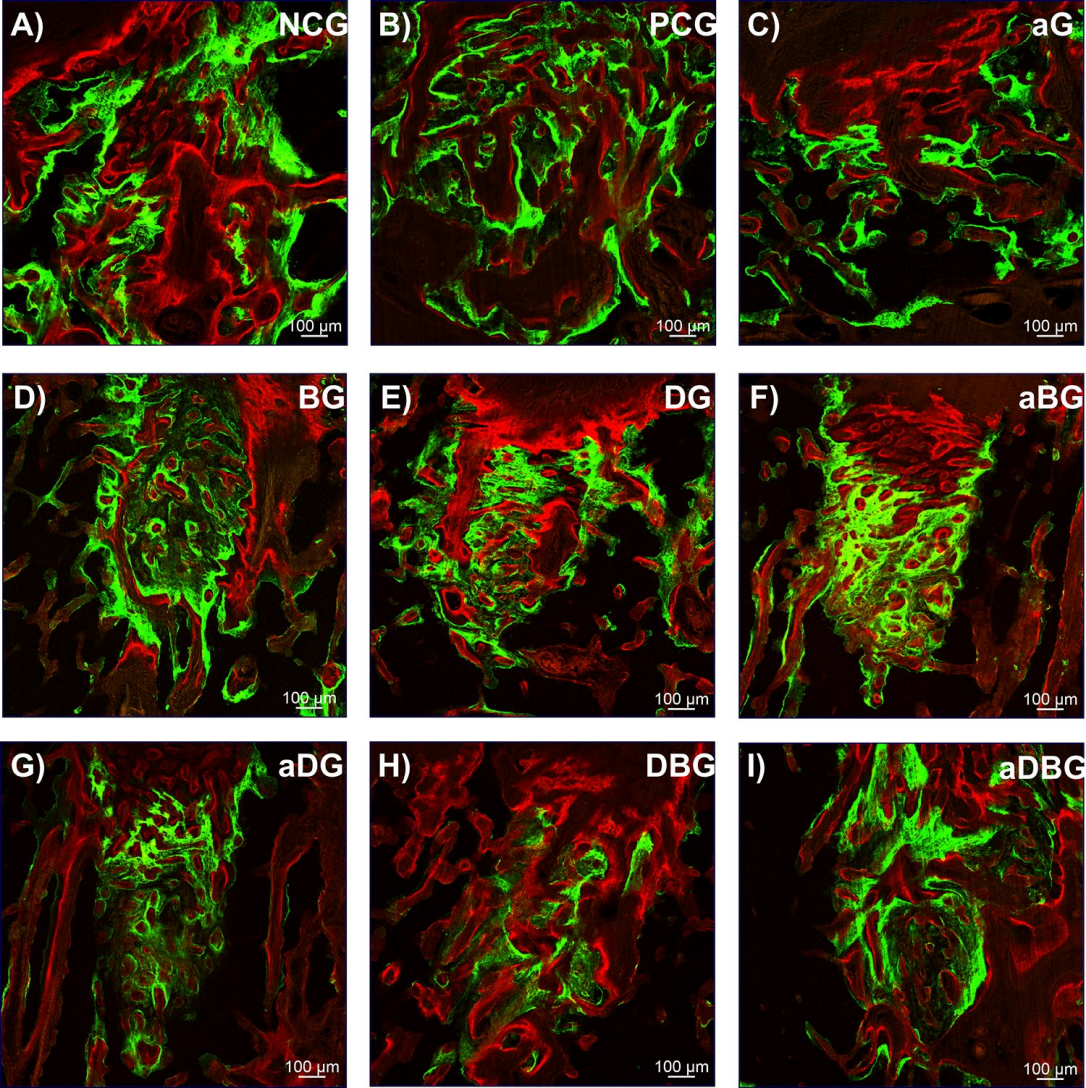
Images representing the dynamics of mineral apposition in the tissue as observed through a confocal laser microscope (10 ×) with calcein fluorochrome (stained in green) and alizarin fluorochrome (stained in red).

Calcium precipitation in the organic matrix with calcein and alizarin, was observed as a fluorescent line in the images. In this study, the fluorochromes were injected 15 (calcein) and 25 (alizarin) days after molar extraction. It stands out that, a green line (calcein) marked the older bone, whereas a red line (alizarin) marked the new bone. Concomitant observation of the images helped differentiate the amount of old and new bone (Fig. 5).

### Histomorphometric analysis

#### Qualitative analysis

**Figure 6:**
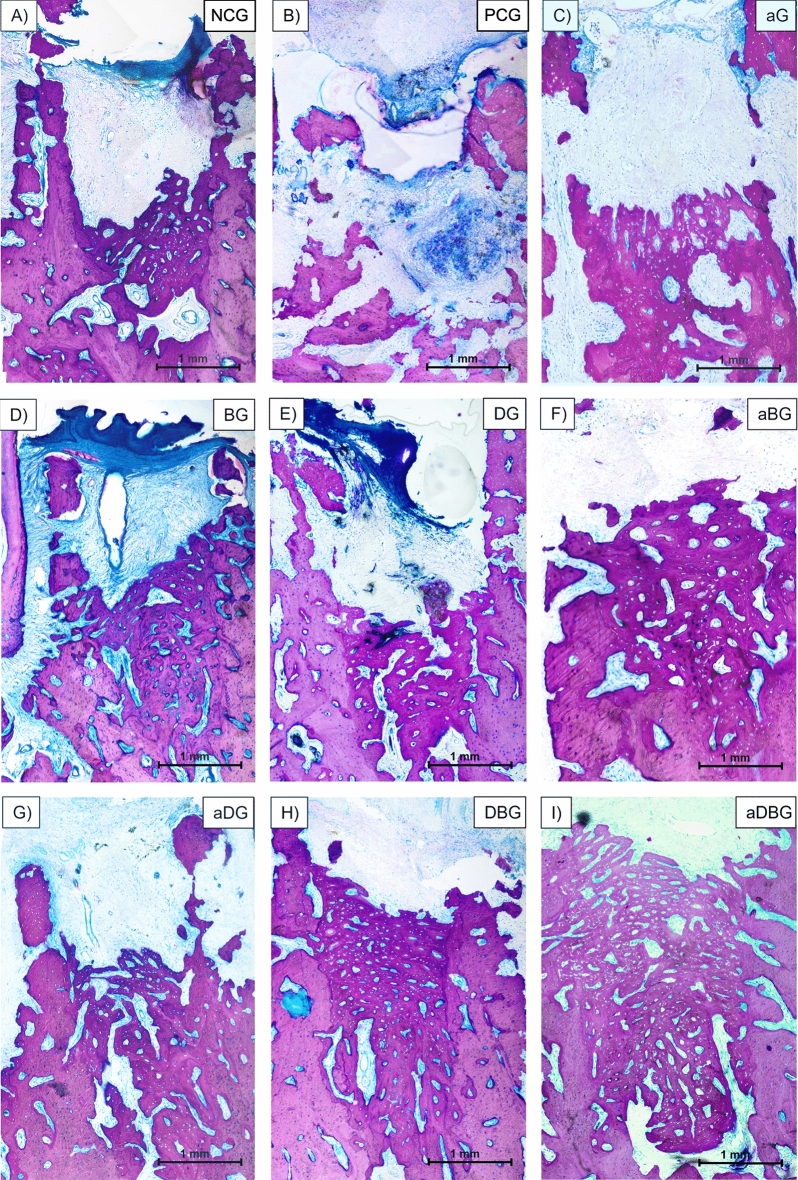
Representative photomicrographs of sections, stained in Stevenel’s blue and acidic fuchsin at 1.6 × magnification (blue, connective tissue; pink, mineralized tissue). Image (**A**) represents the NCG group, (**B**) PCG, (**C**) aG, (**D**) BG, (**E**) DG, (**F**) aBG, (**G**) aDG, (**H**) DBG, and (**I**) aDBG.

**Figure 7:**
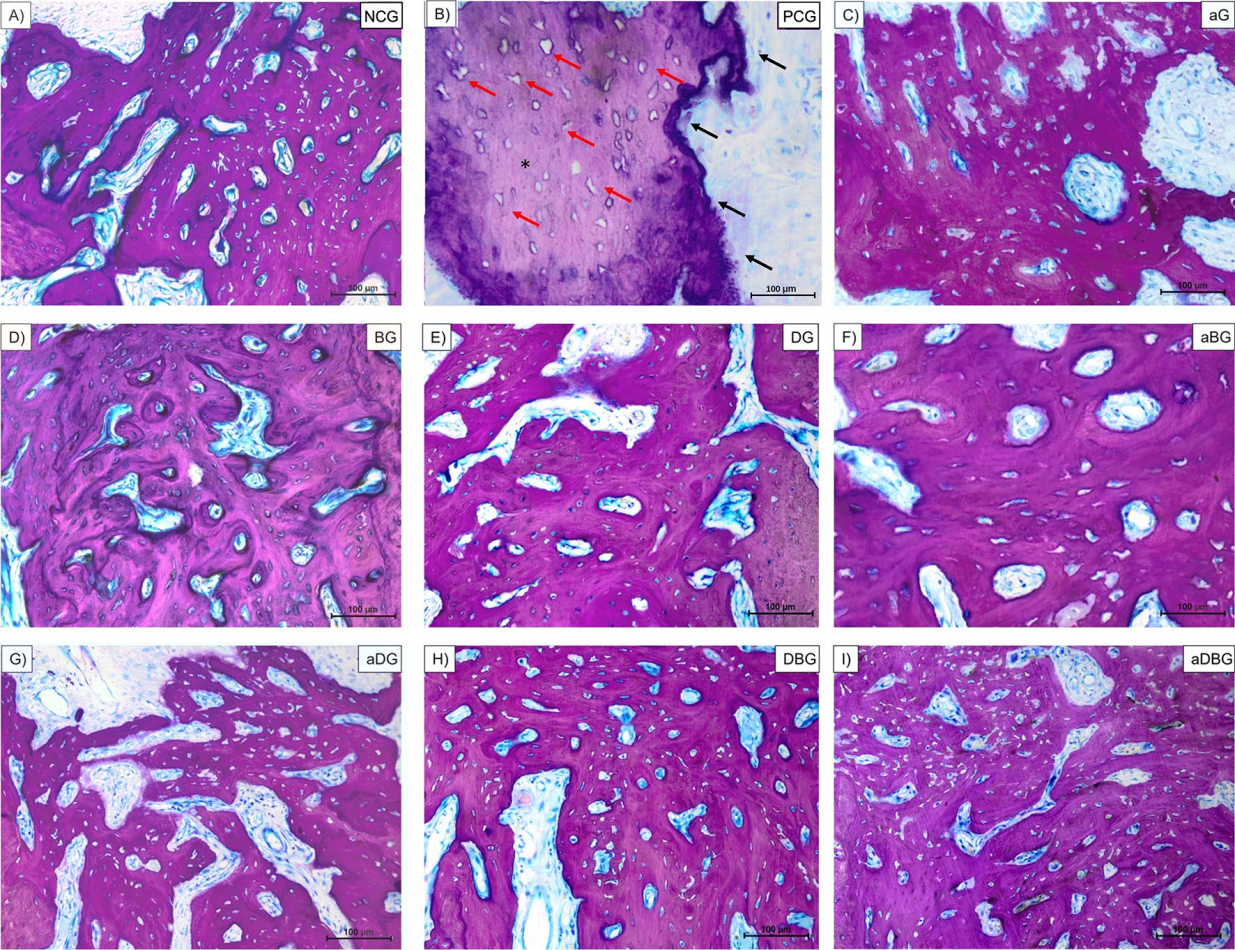
Representative photomicrographs of the sections stained in Stevenel’s blue and acidic fuchsin at 40 × magnification (blue, connective tissue; pink, mineralized tissue). Image (**A**) represents the NCG group, (**B**) PCG, (**C**) aG, (**D**) BG, (**E**) DG, (**F**) aBG, (**G**) aDG, (**H**) DBG, and (**I**) aDBG. (*, bone fragment in the middle of connective tissue; red arrows, empty lacunae without osteocytes; black arrows, irregularities in bone surface).

Analysis revealed that bone formation occurred through a continuous remodeling process involving resorption of old bone and subsequent formation of new bone by osteoblastic action (Figs. 6 and 7). The presence of mature cortical bone (Fig. 6A), along with concentric lamellae, and osteocytes (Fig. 7A) were indicative of the alveolar bone healing in the apical and middle thirds, in the NCG. However, irregularities were observed on the surface of the bony trabeculae in PCG, with the absence of continuity of the cortical layer, and bone fragment loosening, characterizing the bony sequestrum (necrotic bone) (Fig. 6B). At higher magnification, osteocytes and empty Haversian canals were observed (Fig. 7B).

Similarly, the isolated treatments (aG, BG, and DG) allowed bone neoformation in the middle and apical thirds, with foci of vital bone in the connective tissue (Fig. 6C–E, respectively), with a greater amount of bone area in the BG. It can be highlighted that the BG and DG had more trabecular bone (Fig. 7D,E), whereas the aG (Fig. 7C) demonstrated less bone formation.

The combination therapies proposed in this study (aBG, aDG, DBG, and aDBG) were effective in the bone-healing process. Bone neoformation was noted in the apical and middle thirds of the alveolus, in addition to the cervical-third portion, in the DBG and aDBG. aBG (Fig. 6F) and aDG (Fig. 6G) demonstrated more trabecular bone tissue, with several islands of surrounding connective tissue (Fig. 7F,G, respectively), whereas DBG (Fig. 6H) and aDBG (Fig. 6I) showed more organized bone tissue and less connective tissue (Fig. 7H,I, respectively).

#### Bone dynamics

**Figure 8:**
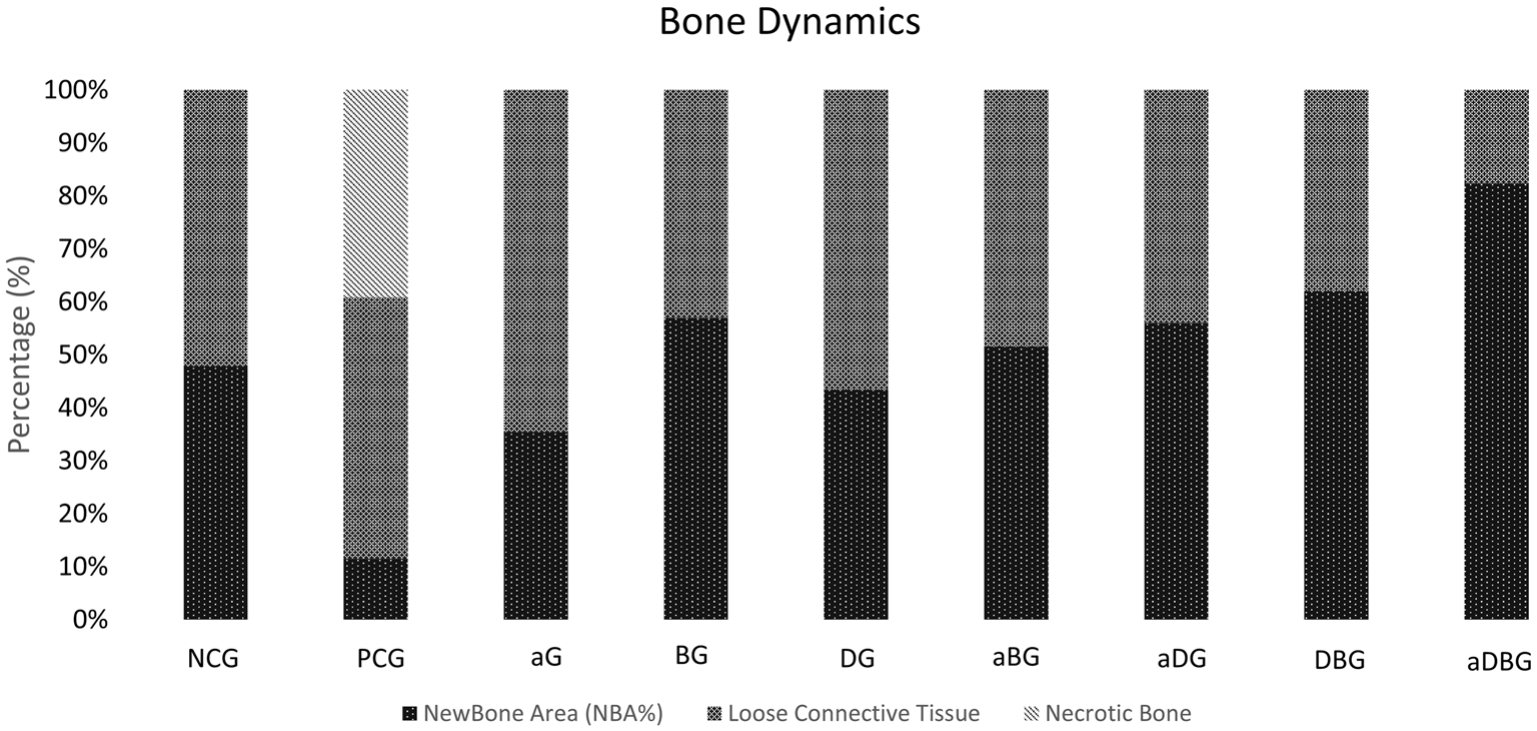
Graph representing the dynamics/composition of the sections, according to each group (in percentage). The components evaluated were neoformed bone area (pink), connective tissue (blue), and necrotic tissue or non-vital bone (gray).

The dynamics of the tissues present within the distal alveolus of the extracted tooth were quantified and organized into (1) %NBA, (2) connective tissue, and (3) necrotic bone (non-vital bone) (Fig. 8); their quantifications and p values are presented in Table 1.

**Table 1:** Summarization of the % NBA and % connective tissue values with theirs respectively significances differences when compared to PCG.

Groups	% NBA	*p* value	% Connective tissue	*p* value
PCG	11.15% ± 7.45		47.78% ± 20.73	
NCG	47.16% ± 4.03	< 0.001	51.36% ± 4.03	> 0.05
aG	35.48% ± 6.58	0.007	64.52% ± 6.58	> 0.05
BG	57.13% ± 5.89	< 0.001	42.86% ± 5.89	> 0.05
DG	41.64% ± 11.46	< 0.001	58.35% ± 11.46	> 0.05
aBG	52.08% ± 10.70	< 0.001	47.92% ± 10.70	> 0.05
aDG	55.90% ± 13.78	< 0.001	44.10% ± 13.78	> 0.05
DBG	60.82% ± 4.37	< 0.001	39.17% ± 4.37	> 0.05
aDBG	82.44% ± 2.69	< 0.001	17.59% ± 2.69	> 0.05

#### Percentage of new bone area (%NBA)

**Figure 9:**
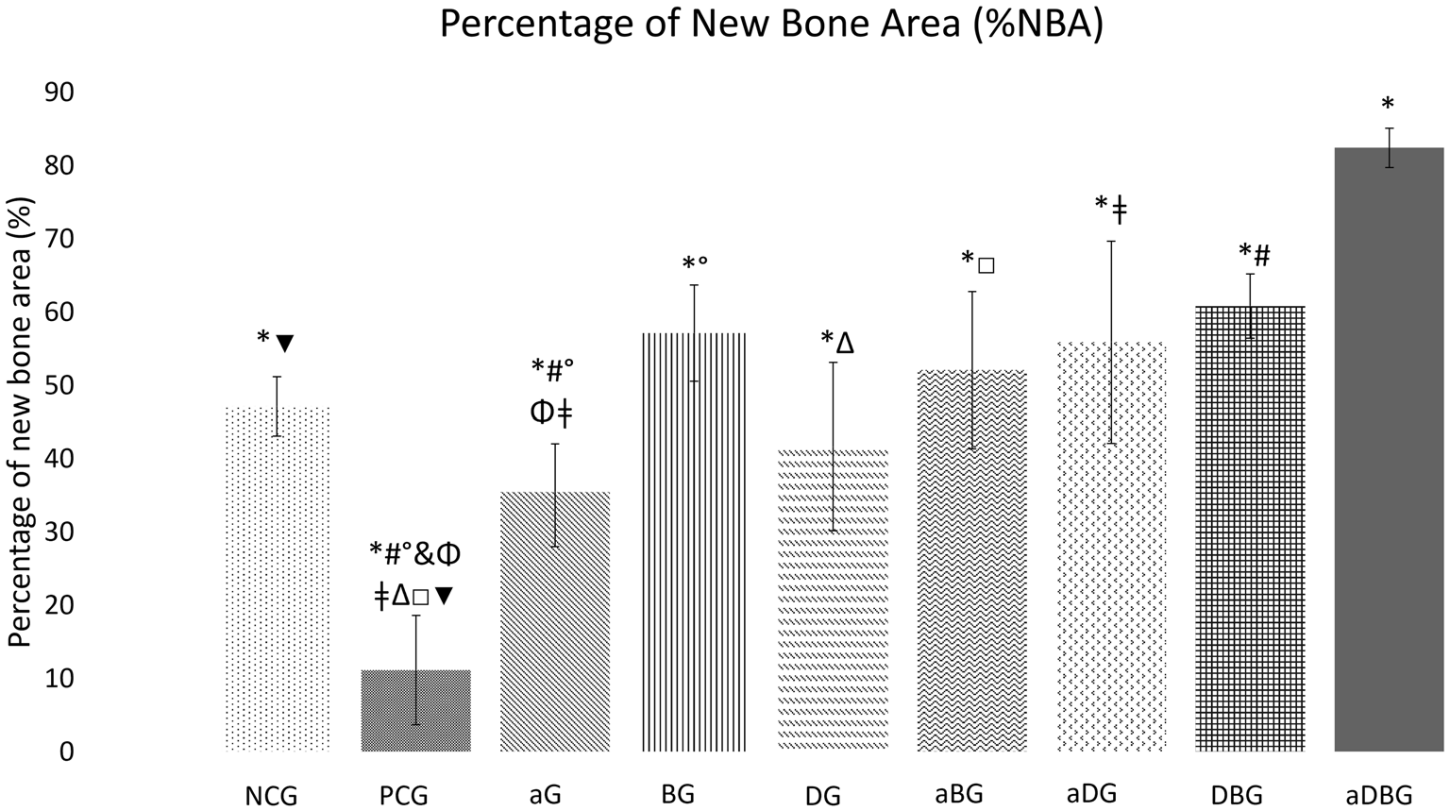
Graph representing the group-wise average percentage of the neoformed bone area (*p* < 0.05, represented by *, when compared to aDBG; #, when compared to DBG; ǂ, when compared to aDG; °, when compared to BG; □, when compared to aBG; Δ, when compared to DG; Φ, when compared aG; ▼, when compared to PCG).

The NCG had a %NBA of 47.16% ± 4.03, whereas the PCG had the lowest %NBA (11.15% ± 7.45; *p* < 0.001) (Fig. 9). The aDBG had the highest %NBA (82.44% ± 2.69) (*p* < 0.05), followed by the DBG (60.82% ± 4.37). The combination therapies aBG and aDG showed satisfactory results for %NBA (52.08% ± 10.70 and 55.90% ± 13.78, respectively), which were different from those of the PCG (*p* < 0.001). Furthermore, the %NBA of aDG was superior to that of aG (*p* = 0.037). Among the isolated treatment groups, the BG showed the best results (57.13% ± 5.89), with significant differences from that of aG (*p* = 0.022) and PCG (*p* = 0.001). The DG and aG showed %NBA of 41.64% ± 11.46 and 35.48% ± 6.58, respectively, which were both superior to that of the PCG (*p* < 0.001 and *p* = 0.007, respectively).

#### Percentage of connective tissue

**Figure 10:**
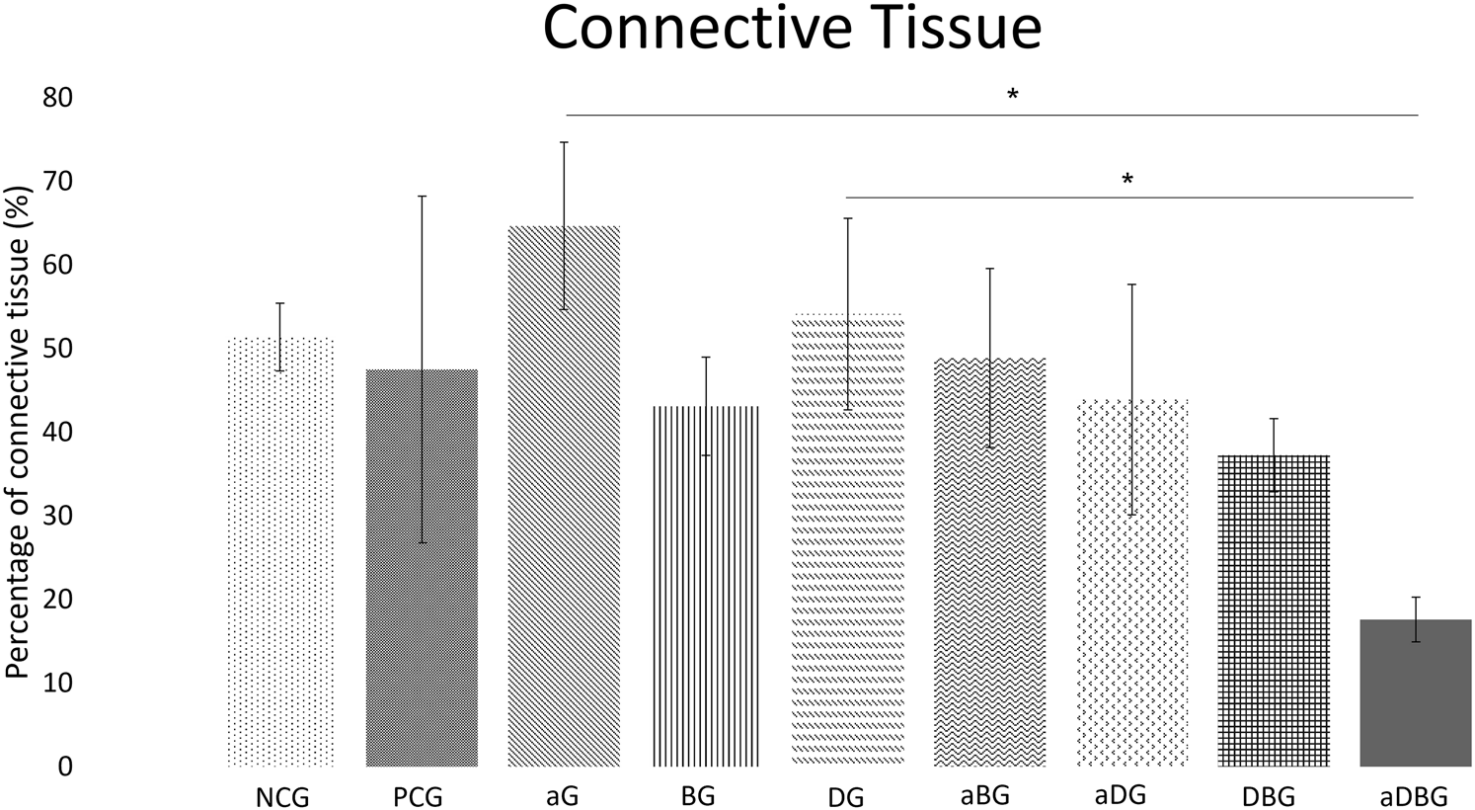
Graph representing the group-wise average percentage of connective tissue area. (*, *p* < 0.05) when compared to aDBG).

The NCG presented with 51.36% ± 4.03 of connective tissue, whereas the PCG presented with a smaller amount (47.78% ± 20.73; *p* = 0.688) (Fig. 10). In contrast to the %NBA, the aDBG had the lowest mean percentage of connective tissue (17.59% ± 2.69), especially when compared to that of the aG and DG (both *p* < 0.05). For the DBG, aDG, and aBG, the percentages of connective tissue were 39.17% ± 4.37, 44.10% ± 13.78, and 47.92% ± 10.70, respectively (*p* > 0.05). The BG had the lowest mean connective tissue among the isolated treatment groups (42.86% ± 5.89), followed by the DG (58.35% ± 11.46) and aG (64.52% ± 6.58) (*p* > 0.05).

#### Percentage of necrotic bone (non-vital bone)

Necrotic bone was not observed in the samples from any of the groups, except for those from PCG (37.94% ± 18.70).

## Discussion

In this study, we hypothesized that local therapies would help prevent MRONJ, thus favoring the bone-healing process. The therapies used here, either alone or in combination, facilitated the formation of vital and organized bone tissue (Figs. 6 and 7). The aDBG, DBG, and aDG exhibited the best results, as demonstrated by the BV/TV through micro-CT analyses (Fig. 2), MAR (Fig. 4), bone dynamics (Fig. 8), and %NBA (Fig. 9); however, the PCG presented 37.94 ± 18.70% of non-vital bone (Fig. 8), embedded in a loose connective tissue stroma (Figs. 6B and 7B).

Precipitation analysis (MAR) represents the amount of bone formed after calcium precipitation in the collagen-bone matrix; owing to the fluorochromes' property of binding to calcium, the amount of fluorescence represents the amount of bone formed^32,33,36,37^. It is noteworthy that BG exhibited the highest response among the therapies applied in isolation (Fig. 4), and among all treatments, the highest MAR was observed in the aDBG (*p* < 0.05). It is believed that the osteoconductive potential of the BTCP-based biomaterial could induce and guide bone formation, providing good results as demonstrated in this research. Furthermore, the aDBG group conglomerated the beneficial effects of each therapy, as demonstrated by MAR.

Excessive suppression of osteoclasts is believed to impair bone turnover, and bone trauma prevents tissue healing, resulting in bone necrosis^16^. Moreover, some BPs, such as ZOL, have antiangiogenic effects that decrease the expression of endothelial growth factors in the circulation, leading to areas of ischemia, facilitating MRONJ development^38,39^. Unfortunately, in this study, the vascularization potential was not assessed; however, it was demonstrated that the use of BPs resulted in the formation of non-vital bone fragments (Fig. 7B), characterizing bone sequestrum (Fig. 3). The use of ZOL allows the tissues to present an intense daily MAR (Fig. 4), reflecting an altered bone function^40,41^.

The presence and viability of fibroblasts and endothelial cells are essential for alveolar bone healing^42–44^. ZOL can inhibit osteoclast formation, alter cell migration, suppress angiogenesis and osteogenesis, thus generating toxicity in these cells^45–51^. Inhibition of these cells encourages the formation of a more dense, cortical bone, instead of a vascularized tissue (Fig. 6B), thus increasing the likelihood of bone sequestration associated with inflammation, and consequentially, local infection^52,53^, as demonstrated in the PCG (Fig. 7B).

Animal models have been used to demonstrate the repercussions of BPs on bone, such as an increase in bone density and a decrease in the spaces between the trabeculae, causing a decrease in the nutritive channels of the bone tissue^30,54–56^. In the present study, the region corresponding to the distal alveolus of the first molar was evaluated, and no suggestive results of an increased bone density were found (TbTh, *p* = 0.792; TbN, *p* = 1.901; TbSp, *p* = 0.199; and PoTot, *p* = 0.109). However, in the micro-CT analysis using the NRecon tool in the PCG, areas of bone irregularities, sequestration, and absence of bone within the alveolus were observed, as demonstrated in previously published studies on animals treated with ZOL^20,57–61^.

Recently, an in vitro study demonstrated that the use of calcium phosphate-based synthetic ceramics in a fibroblast culture exposed to ZOL therapy reduced or prevented toxicity^26^, thus favoring alveolar bone healing, as observed in the present study in BG, DBG, and aBG, as %NBA (Fig. 9) and BV/TV (Fig. 2D). Ceramics-based biomaterials composed of BTCP are highly biocompatible and bioactive, in addition to being osteoinductive^62–64^, and are thus indicated for filling bone cavities^65^. Studies have shown beneficial effects on cell growth, and these biomaterials can be considered as good candidates for bone tissue engineering applications^66,67^, in addition to demonstrating ectopic bone formation even in large animal models, with a rapid bone formation pattern as that of autogenous bone^68^. The use of ceramic biomaterials based on BTCP^69^ or borate bioactive glass^70^ can help prevent MRONJ and enhance vital bone tissue formation.

The multi-session aPDT in newly extracted alveoli of senile rats treated with ZOL, has prevented MRONJ^24^, as demonstrated in this study (Fig. 3). Biomodulation with a low-power laser has biostimulatory effects on osteoblasts, even when treated with ZOL^27^; the association with a photosensitizing agent (methylene blue) reduces the bacterial load^71^, thus preventing MRONJ, as observed in this study in aG (Fig. 7C).

Infection control strategies, ranging from topical to systemic antibiotics, are essential in the treatment of MRONJ^17^. Several antimicrobial combinations serve as adjuvant therapies. Doxycycline, a structural isomer of tetracycline, has antibacterial, anti-inflammatory, and anti-collagenase activities; its anti-osteoclastogenic potential is essential for bone healing^72–75^. The application of a collagen sponge with doxycycline to the alveolus, after treatment with ZOL, showed positive effects on the reduction of necrotic bone tissue in rats^28^. Similarly, in this study, the use of 10% DG prevented MRONJ (Fig. 3), and contributed to the maintenance of bone volume (Figs. 6E and 7E).

Further, the combination of local treatments showed good results by their synergistic effect, thus satisfying the primary objective of this study to prevent osteonecrosis (Fig. 8). Even though the isolated use of aPDT (aG) has not demonstrated large amounts of bone in %NBA analysis, the authors believe that its antimicrobial and biostimulatory potentials are essential for the prevention of osteonecrosis, and when associated with doxycycline (aDG) and a biomaterial (aBG), a greater amount of bone volume was observed in %NBA analysis. The use of 10% DG alone showed good results in repairing critical bone defects in rat calvaria^76^. It is believed that the anti-osteoclastogenic effect of doxycycline allows the biomaterial to maintain its osteoconductive function. Thus, the combination of the three local methods (aDBG) prevented MRONJ (Fig. 3), favoring alveolar bone healing (Figs. 9I and 10I) with higher %NBA, BV/TV, and MAR.

The mechanisms of alveolar bone healing are similar among species^77^, and rodent models are ideal for assessment as there is no evidence of superiority for the larger animal models in representing human bone biology^78^. Given the methodology and results demonstrated in this study, we believe that the use of a low-dose concentration of ZOL in this study (0.035 mg/kg) could be a limiting factor; it is similar to that used for preventing osteoporosis^30,79^, unlike the high-dose concentrations used in cancer treatment^23,24^. In addition, it can be noted that the choice for male rats could be a limitation in this research, since in translational clinical application, women are more likely to develop osteoporosis, owing to menopause and estrogen deficiency. Further, they pose a higher risk of developing osteonecrosis with the use of antiresorptive drugs to prevent bone loss. Therefore, future studies are necessary to evaluate different concentrations of ZOL, as well as methodologies for the evaluation of demineralized tissues such as immunohistochemical analysis and real-time polymerase chain reaction.

## Conclusion

This research reveals that MRONJ induced by the ZOL protocol (0.035 mg/kg) is counteracted by the use of beta tricalcium phosphate, either alone or in combination, by guiding alveolar bone healing. While the use of doxycycline and aPDT demonstrated positive effects on MRONJ prevention, the combination of all three therapies proved satisfactory.

## Data Availability

The datasets generated and/or analyzed are available from the corresponding author upon reasonable request.
